# Feasibility of using quantitative ^1^H-NMR spectroscopy and ultra-microbalances for investigation of a PET microplastic reference material

**DOI:** 10.1007/s00216-023-04567-0

**Published:** 2023-02-08

**Authors:** John Seghers, Marcel Günther, Andreas Breidbach, Nadine Peez, Wolfgang Imhof, Håkan Emteborg

**Affiliations:** 1 Joint Research Centre (JRC), European Commission, Geel, Belgium; 2 University Koblenz, Koblenz, Germany

**Keywords:** Reference material, Metrological traceability, Microplastics, Characterisation, Quantitative NMR spectroscopy, Gravimetry, Water, Poly-Ethylene terephthalate

## Abstract

Here, we report on the feasibility of using quantitative NMR and ultra-microbalances for additional measurements of the mass of poly-ethylene terephthalate (PET) particles in a reference material (RM). The microplastic (MP) PET particles were immobilised in solid NaCl following freeze-drying of a 1-ml NaCl suspension. The particles ranged from 30 to about 200 µm (Feret_min_). In a 3-day process, more than 500 such units of PET particles in the NaCl carrier were prepared and later used in a large-scale inter-laboratory comparison. The homogeneity of PET in the salt carrier over these 500 units had previously been evaluated with respect to the mass of PET using an ultra-microbalance. In addition to the original results obtained by weighing, two independent results of quantitative ^1^H-NMR have been obtained for further investigation of this reference material together with one additional set of weighing data. The NMR data were used for confirmation of the weighed amount of PET (as weighing is non-specific for PET). Average masses of 0.293 ± 0.04 mg and 0.286 ± 0.03 mg of PET were obtained using two different ultra-microbalances (14% RSD for *n* = 14 and 9% RSD for *n* = 4, respectively). The corresponding ^1^H-NMR data was 0.300 ± 0.02 mg of PET (6.7% RSD for *n* = 5) and 0.345 ± 0.04 mg of PET (12.5% RSD for *n* = 14), respectively. The average mass of PET obtained by ^1^H-NMR measurements was in agreement with the weighed amounts within their standard deviations. A mean value of 0.306 mg PET with an expanded uncertainty of 0.058 mg (± 19% relative) was calculated, and it is traceable to the SI system of measurements. Measurement of PET by quantitative ^1^H-NMR spectroscopy is also reported for a water sample. The PET contained in one RM sample was transferred to 1 L of water to mimic a drinking water sample for microplastics.

**Graphical abstract:**

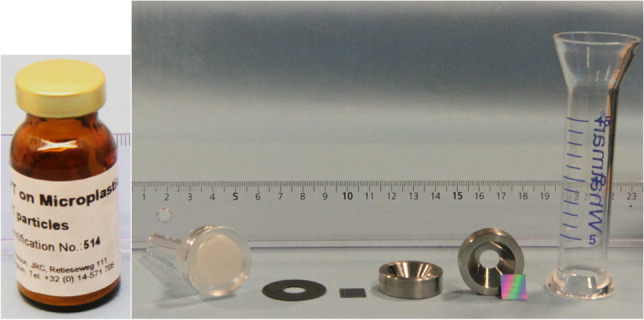

**Supplementary Information:**

The online version contains supplementary material available at 10.1007/s00216-023-04567-0.

## Introduction

Environmental contamination with microplastics (MP) is an increasing problem at global scale as the quantities of plastics entering rivers, seas, agricultural soils and sediments are ever increasing [1]. Frias and Nash proposed a definition for microplastics, namely, “Microplastics are any synthetic solid particle or polymeric matrix, with regular or irregular shape and with size ranging from 1 µm to 5 mm, of either primary or secondary manufacturing origin, which are insoluble in water” [2]. Secondary MP particles, which are the main source of MP, are thus generated by degradation of larger plastic objects that are spread due to poor or inexistent waste management [1].

There is growing evidence that MP could also present a risk to human health since presence of MP in human blood, breast milk and lung tissue has recently been demonstrated by Leslie, Liu and Jenner et al. [3–5].

Under all circumstances, accurate and comparable measurement results are crucial for a deeper understanding and better mitigation of the problems associated with MP. If, for example, successful monitoring of trends of microplastic contamination shall be established, measurement methods must be validated and the measurement results must be traceable to a common point of reference applying the approaches given in ISO/IEC 17025:2017 and the Eurachem Guide for method validation [6, 7]. To help ensure analytical quality assurance, reference materials (RMs) and certified reference materials (CRMs) are efficient tools in the analytical laboratory but with different scopes, which have important implications. The terms RM and CRM are defined in ISO Guide 30 [8]. Consequently, a reference material is a *material*, sufficiently homogeneous and stable with respect to one or more specified properties, which has been established to be fit for its intended use in a measurement process. A certified reference material is a *reference material* characterised by a metrologically valid procedure for one or more specified properties, accompanied by an RM certificate that provides the value of the specified property, its associated uncertainty and a statement of metrological traceability [8].

A previous paper describes the preparation of the microplastic RM studied further in this work [9]. That RM was deemed to be sufficiently homogeneous and stable to be used in a large-scale inter-laboratory comparison (ILC), but it was lacking an assigned value. Only a conservatively estimated indicative range was provided to the ILC participants after the exercise. This RM is an example of a non-certified reference material. All ILC results using this reference material are summarised in a comprehensive technical report from the Joint Research Centre of the European Commission [10].

A non-certified reference material still has meaningful use, e.g. to prepare control charts or for use in proficiency testing. It is useful for relative comparisons, as shown using the MP PET material at hand [9–11]. In the case of CRMs, they can also be used for method validation, a trueness check and provision of metrological traceability of the measurement results, which is the basis for accurate measurements providing comparability over time and between different laboratories [6, 7, 12, 13].

When producing a CRM, the characterisation step is a key process of assigning certified property values to a reference material (RM). For a quantitative parameter, an accurate property value can be achieved by formulation applying accurate and precise weighing of ingredients of known purity. An inter-laboratory comparison using two or more analytical methods of demonstrable accuracy in one or more competent laboratories is another way to characterise an RM or by using a combination of approaches [12, 13]. For this reason, it is useful to explore accurate measurement techniques that can be used for characterisation of reference materials for microplastics.

A series of papers have demonstrated the suitability of ^1^H-NMR for specific quantification of microplastics like PET and other polymers [14–16]. In the current work, ultra-microbalances and quantitative ^1^H-NMR spectroscopy were used to further investigate the amount of PET in a microplastic reference material [9]. Quantification of PET using ultra-microbalances is non-specific because any solid material collected on the dried silicon filters (which were used in these studies) contributes to the mass as previously discussed in the paper by Seghers et al. [9]. Therefore, to confirm the mass of PET obtained by weighing, ^1^H-NMR was used for quantification of microplastics in remaining units of this reference material. Good agreement between the two different measurement approaches would confirm absence of any major contribution from other particles than PET in the RM as already shown by optical microscopy in the previous study and to establish an accurate mean value of PET in this material.

As far as the authors are aware, there are currently no certified reference materials available for quantitative parameters in the field of microplastic measurements, e.g. where the number of MP particles or mass of MP is certified.

The current work was performed to demonstrate the feasibility of measuring PET in remaining units of the reference material used in the ILC using a combination of techniques [9, 10]. The long-term stability of PET microplastic particles in the salt matrix was also established over a period of 31 months, which together with the homogeneity and additional measurements of PET, was used to establish the expanded uncertainty of the mean value given as mass of PET in the salt carrier.

The concept and approaches developed for the work reported here pave the way for MP (C)RM preparations currently under way at JRC-Geel.

## Experimental

### ^1^H-NMR at University Koblenz

#### Sample preparation for salt carrier RM

Three millilitres of deionised water was added to the RM units to dissolve the salt carrier. Afterwards, 1 mL of an extractant, consisting of 80% chloroform (CHCl_3_,  >99% purity, Carl Roth GmbH & Co. KG, Karlsruhe, Germany) and 20% trifluoroacetic acid (TFA,  >99.9% purity Carl Roth) (v/v), was added. The closed vessels were shaken by hand for 5 min, achieving phase separation within another 5 min standing still after shaking. By use of a hypodermic syringe, the extract was collected in a separate vial and the extraction cycle was repeated four times. In total, 5 mL of extract was collected and evaporated using an air stream at 60 °C during 45 min. As NMR solvent, a mixture consisting of 80 parts CDCl_3_ of 99.8% purity (DEUTERO GmbH, Kastellaun, Germany), 20 parts TFA, 0.1 part hexamethyldisiloxane (HMDSO) of 98% purity (Carl Roth) and 0.1 part dimethyl sulfoxide (DMSO) of  >99.5% purity (Carl Roth) was used (v/v). The solid residue obtained after the sample preparation was then reconstituted in 1 mL of the NMR solvent.

For external calibration, five samples with concentrations of 0.1, 0.2, 0.3, 0.4 and 0.5 mg/mL were prepared by dilution of a PET stock solution [15].

#### NMR measurements

For the NMR measurements, 0.7 mL of the resulting solution was added to a 5-mm NMR tube closed by a PTFE cap. Measurements were performed on a Jeol® 500 spectrometer with a 500-MHz 5-mm TH ATM probe-head at room temperature. All samples were measured three times using the following parameters: ^1^H-NMR; angle: 90°; scans: 25; acquired size: 32,768; spectral width: 11 ppm; receiver gain: 36; relaxation delay: 25 s; and acquisition time: 5.95591 s.

#### Data analysis

For data analysis, the software MestReNova was used [17]. The chemical shift was referenced to the CHCl_3_ signal at 7.25 ppm. Phase and baseline correction were performed manually. Apodization was set to 0.5 Hz. Signals were integrated in the following regions: DMSO at 2.75 to 3.10 ppm, PET (aliphatic) at 4.67 to 5.00 ppm and PET (aromatic) at 8.00 to 8.25 ppm.

Further calculations including the normalisation of signal intensities as well as linear regression were performed according to the method described by Peez et al. [15]. DMSO was used as an internal standard for normalisation. Precision was calculated as the relative standard deviation for each RM sample based on three replicate measurements. The limit of detection (LOD) and limit of quantification (LOQ) are based on the signal-to-noise ratio of 3 and 10, respectively, and reported in Table S1.

#### Sample preparation for a water sample spiked with the salt-carrier RM

The content of one salt-carrier RM sample was transferred into a 1-L glass bottle containing 950 mL of filtered water. To ensure a complete transfer of the PET, the sample vial and insert were checked for any particles (or salt) that were attached to the inside of the Teflon-coated insert. Afterwards, the salt carrier and PET particles were dissolved in 5 mL water and poured into the 1-L glass bottle. This procedure was repeated eight times. Additionally, the insert of the vial was finally rinsed with 5 mL of water directly into the 1-L glass bottle. The accumulated rinsing portions of 50 mL and the 950 mL of water made up a final sample volume of 1 L of water. After brief mixing, the water sample with MP was filtered through a P4-glass frit and washed with deionised water. The solid particles on the frit were transferred in two steps into a 20-mL glass vial: first manually by using a spatula and then by rinsing with small portions of water until the vial was almost filled. Afterwards, the water was evaporated overnight at 60 °C in a drying cabinet that was exclusively used for MP samples. Finally, the solid residue was dissolved in 1 mL of CDCl_3_/TFA (4:1), using 0.7 mL for measurements by NMR.

### ^1^H-NMR at JRC-Geel

#### Sample preparation

Quantitative NMR was also performed at JRC-Geel using an internal reference standard instead of external calibration. The internal reference standard used for these quantitative NMR measurements is dimethyl sulfone (DMSO_2_) certified by NMI Australia with a mass fraction purity of ${w}_{S}=1000 \pm 0.8\mathrm {\ mg}/\mathrm{g}$ with the uncertainty representing the half width of a 95% confidence range.

The PET particles were extracted by liquid–liquid extraction with some slight modifications compared with the approach described above. Three millilitres of type-1 water was added to an RM sample [18]. With gentle agitation, the salt carrier was dissolved. Once a clear solution was obtained, 1 mL of CHCl_3_/TFA (4:1, v/v) was added and the unit was shaken vigorously by hand. Both CHCl_3_ and TFA were of HPLC grade and obtained from VWR, Belgium. Then, the unit was agitated in a wrist action shaker at 70 Hz for another 5 min. To facilitate phase separation, the unit was centrifuged for 1 min at a relative centrifugal force of 3200 × *g*. The organic layer was removed from the bottom with a glass syringe and transferred to a clean glass tube. Addition of organic solvent, agitation, centrifugation and transfer were repeated three additional times and collected in the same glass tube for a total volume of PET solution of about 4 mL. The solvent was then evaporated in a vacuum centrifuge at 40 °C to complete dryness. To the dry residue, the internal reference DMSO_2_ was added on a microbalance (*d* = 0.001 mg).

This blend was finally dissolved in 1 mL CDCl_3_/TFA (4:1, v/v). After 5 min, 600 µL was transferred to a 5-mm NMR tube. The deuterated chloroform (CDCl_3_, NMR grade containing 0.03% trimethylsilylpropanoic acid) was obtained from VWR, Belgium.

#### Measurements

All measurements were executed on a Bruker Ascend 400 with a 5-mm PABBI 1H/D-BB Z-GRD Z820201/0276 probe-head and a “Sample Express” sampler changer. Acquisitions were controlled using the Bruker’s Topspin V3.2 software. After two dummy scans, 100 scans were acquired per measurement with pulse program “zg” at a scan width of 40 ppm and a free induction decay (FID) size of 128,202. The scan delay time was set to 43 s to obtain full relaxation between scans, and the pulse width was set to 8.12 µs for a 90° pulse.

Measurement batches were run by “IconNMR”. Per NMR tube, two of the above measurements were executed with shimming for each new tube automatically done with the “topshim tunea” procedure.

#### Evaluation of NMR data

NMR data were evaluated with the statistical software suite “R V4.1.2”. The functions of package “PepsNMR V1.12.0” were used to convert the free induction decays (FIDs) to the frequency-domain spectra. An apodization with a line broadening of 0.3 Hz was used to improve the signal–noise ratio. The frequency-domain signal was baseline corrected with the “baseline” function of R’s “FTICRMS V0.8” package. The DMSO_2_ signal at 3.16 ppm (6H, s) and the signal of the aliphatic protons in the PET repeating units (4.79 ppm, 4H, singlet) were integrated in a range from 10 times the full peak width at half maximum (FWHM) to 10 times FWHM taken from the peak apex. The integration range was limited to 10 times FWHM to not include the signal of the PET end group protons. The integral of the PET signal was divided by the integral of the DMSO_2_ signal to obtain the ratio, *R*. The two measured ratios *R* per NMR tube were averaged for the following calculations.

The measurement equation to assign a mass amount to the PET with NMR is as follows:

1 $${m}_{\mathrm{A}}=R\mathrm{^{\prime}}\frac{{N}_{\mathrm{S}}}{{N}_{\mathrm{A}}}\frac{{M}_{\mathrm{A}}}{{M}_{\mathrm{S}}}\frac{{m}_{\mathrm{S}}}{REC}{w}_{\mathrm{S}}$$

with $R\mathrm{^{\prime}}$ being the average of all ratios *R* per test unit, $N$ representing the number of ^1^H nuclei contributing to the integrated signals, $M$ representing the molar mass, $m$ representing the mass and $w$ representing the mass fraction purity. The indices $A$ and $S$ stand for analyte and internal standard, respectively. The assumption for Eq. 1 to be correct is that the mass fraction purity of the PET added to the salt carrier equals one. A molar mass of 192 g/mol of the PET repeating unit is used for *M*_A_*.* For *M*_S_, the molar mass of DMSO_2_ (94 g/mol) is used. The term *REC* corrects for the overall recovery of the extraction of the PET from the salt matrix determined from independent experiments during a method validation study. Measurement uncertainty was estimated top-down from validation data establishing relative repeatability standard deviation as well as intermediate precision RSD and is reported in Table S2.

### Use of macroporous silicon membranes for weighing of PET using ultra-microbalances at JRC-Geel

The original weighing approach made use of macroporous silicon membrane filters of 1 × 1 cm, pore size of 5 µm with a pore inter-distance of 12 µm and 500 µm thickness. These filters were obtained from Smart Membranes, Halle, Germany, and used as described in previous work using the UMX-5 balance [9]. In the initial set-up, a rubber ring, a glass recipient of 20 mL volume and a white Teflon ring was used. The white Teflon ring was not completely flat, and its colour made it hard to see remaining PET particles visually.

### Slightly adapted weighing approach

For subsequent filtrations of the PET suspensions obtained from the RM sample, each 1 × 1-cm square filter was placed on a rubber sealing ring placed on the glass frit of the filter holder. The filter was placed on the rubber ring with the shiny filter side facing upwards. The filter holder was connected to a vacuum pump through a vacuum flask. Thereafter, a custom-made stainless steel funnel and the glass recipient of 20 mL was attached with a metal clamp directly onto the filter resting on the rubber ring and glass frit of the filter holder. The stainless steel funnel has an inner diameter of 8 mm at the bottom and 20 mm at the top with a depth of only 8 mm. It therefore resembles a ring more than a funnel and it is conical towards the bottom to minimise potential losses of PET particles (see Figure S1 for pieces used for filtering). The NaCl carrier with PET was dissolved and transferred using ten portions of 5 mL 0.1% Triton X-100 surfactant poured directly in the vial, which was then swirled and emptied onto the filter under suction. Afterwards, the vial was rinsed thoroughly several times with type-1 water to transfer all particles to the filter. The filter was thereafter rinsed with type-1 water to remove any residual Triton X and NaCl [18]. A final rinse with 5 mL of ethanol was performed to make sure all particles were transferred and to facilitate drying at 60 °C. It is important that the sample and solutions are kept at room temperature before filtration to avoid condensation on the stainless steel funnel after filtration. Before removing the stainless steel funnel, the funnel should be tapped gently with a spatula to release particles sticking to the metal funnel. The stainless steel funnel was then removed and inspected for any remaining particles present on the edges. If necessary, such particles can be brought back on the filter with a needle. All filtering took place in a Nuaire NU-164 clean bench (Plymouth, MN, USA). Thereafter, the filters were dried at 60 °C overnight in a petri-dish with a slightly open lid using an oven (Carbolite, LHT5/120, Hope, UK).

Weighing of PET captured on silicon filters was performed using two different ultra-microbalances. The first balance used was the UMX5 model (Mettler-Toledo AG, Greifensee, CH) using the principle of substitution weighing as already described in previous work [9]. The JRC report also contains a dedicated fact box about substitution weighing [10]. That balance has a maximum load of 5 g with 0.1 µg resolution. The second ultra-microbalance was a UMT2 model (Mettler-Toledo AG, Greifensee, CH) which was subsequently used without applying substitution weighing. This balance has a maximum load of 2 g with a 0.1 µg resolution. Instead, a reference weight of 1 mg class E2 was weighed before and after every weighing exercise to ensure adequate performance of the balance. No further corrections for air pressure, relative humidity and temperature were made.

## Results and discussion

### Discussion (NMR—external calibration/internal calibration)

The quantification of substances using NMR can be performed using two main routes, using an internal reference standard (JRC-Geel) or external calibration (University of Koblenz). These approaches also comprise diverse normalising methodologies and ways of integration or characterisation of functional groups.

In the case of the external calibration method, normalised signal intensities of unknown samples are first computed according to the procedure described in the “Data analysis” section. These normalised signal intensities are subsequently used to calculate the PET content by interpolation of a linear regression function obtained from an external calibration curve.

In order to achieve sufficient recovery rates, multiple extraction cycles are necessary for both sample preparation methods regardless of calibration mode. This need is caused by the extractant used since TFA is soluble in both phases. A major fraction of TFA presumably distributes from the organic chloroform phase into the water phase during the extraction. This reduces the amount of TFA in the extractant, which can lead to re-precipitation of PET during the initial extraction cycles, as observed in some cases. It can be assumed that deviations between the analysed samples are mainly caused by the extraction process rather than by the NMR measurements themselves. This assumption is supported by the high precision of repetitive measurements of the same sample (Table S1).

For the quantification using external calibration, hexamethyldisiloxane, HMDSO, was initially used as an internal standard since signal overlaps with other substances are unlikely. Nevertheless, measurements of the PET RM units after extraction without the addition of internal standard displayed several undesired signals that would overlap with the HMDSO signal. These signals are presumably residues of Triton X-100 deriving from the preparation of the samples and co-extract during sample preparation [9]. Therefore, HMDSO had to be replaced. An alternative internal standard was therefore selected. Dimethyl sulfoxide, DMSO, was chosen as its boiling point of 189 °C ensures a low vapour pressure lowering the risk for concentration changes during sample preparation and measurements. Furthermore, DMSO shows only one singlet at 2.92 ppm, which is not overlapping with any other signals of the RM PET sample. In Figure S2, respective spectra can be compared proving the suitability of DMSO as an internal standard added to the NMR solvent for signal normalisation.

The external calibration approach is best utilised if high throughput of samples is intended. At University Koblenz, the internal standard was added to the solvent only to compensate for fluctuations of the NMR instrument. Contrary to the internal reference standard approach applied by the JRC, detailed knowledge of signal shape and associated proton number or precise selection of integration areas is not as important as long as interferences with matrix signals or impurities can be excluded. Instead, only an accurate repetition of data treatment and constant measuring parameters must be ensured. Thus, external calibration can be applied easily without excessive knowledge of NMR analysis.

On the other hand, gravimetric addition of an internal reference standard for quantification is best suited if only a handful of samples have to be analysed and high accuracy is required. Even though the internal reference standard must be added to every sample, there is no need for additional calibration to allow subsequent quantification. Thus, each sample can be analysed independently from other measurements.

### Mass of PET in the water sample

**Fig. 1:**
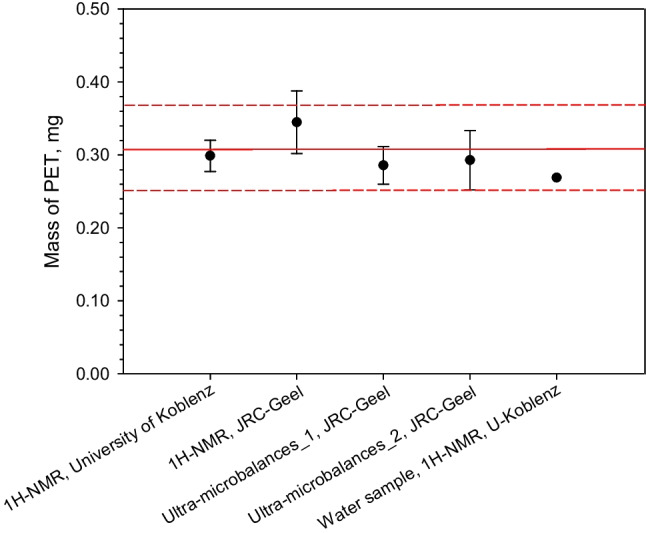
The solid red line shows the mean value of 0.306 mg of PET in the RM. The dashed red lines show the ± expanded uncertainty of 0.058 mg. The data sets obtained using ultra-microbalances and ^1^H-NMR are shown with their respective mean values ± one standard deviation. To the right, the result of measuring PET by spiking one unit of the RM into 1 L of water shown as a single data point

The mass of PET in the water sample was determined by a single measurement using the integral of the aromatic PET signal in the region of 8.00–8.20 ppm according to the method of Peez et al. [15]. That single result was reported as a part of the inter-laboratory comparison described in the technical report from the JRC [10]. Each participant to the ILC only had access to two units whereof one sample was for practicing. The result of the second sample was to be reported. Hence, that single result was 0.269 mg/L. It falls within the expanded uncertainty of the mean value shown in Fig. 1. This result shows the usefulness of the salt carrier for spiking and that it is indeed possible to add the RM to a matrix that is relevant for studies of microplastics, e.g. drinking water.

### Comparison of results for PET obtained by ^1^H-NMR and weighing using ultra-microbalances—calculation of a mean value for all data sets

**Fig. 2:**
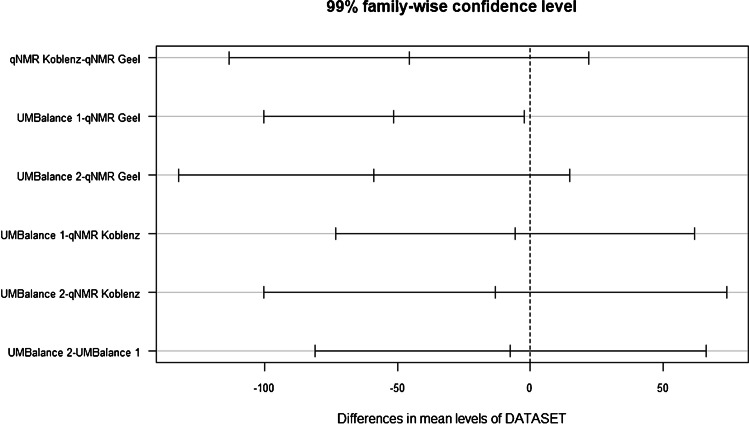
Graphical representation of Tukey’s range test. Each horizontal line with the three vertical marks shows the difference to the overall mean (centre mark), and the lower (left) and the upper (right) end of the confidence range of the respective comparison. On the *x*-axis, the differences in mean levels of DATASET are given in micrograms. Overlap with the dashed vertical line at 0 means that there is no statistically significant difference between the results at a 99% confidence level. UMBalance denotes ultra-microbalance

Two data sets from ^1^H-NMR and two data sets using ultra-microbalances comprising measurements from 37 different RM units were available. This number corresponds to more than 7% of the total number of units prepared. Before combining the four data sets obtained by weighing and measurements by q-NMR, a Tukey’s range test was applied to verify that the mean values of the data sets were all coming from the same population [19]. The Tukey test is a single-step multiple-comparison procedure and statistical test. It is used to find means that are significantly different from each other. Before applying the test, three assumptions must be fulfilled. The observations being tested must be independent within and among the groups, the groups associated with each mean in the test must be normally distributed, and there is an equal within-group variance across the groups associated with each mean in the test. A confidence level of 99% was chosen to minimise the false exclusion of one or more of the data sets. Only the comparison of the data sets from q-NMR Geel and ultra-microbalance 1 are different according to this statistical test. Figure 2 depicts the results graphically, and the outcome is further explained in the figure caption.

Nevertheless, even if the difference is statistically significant, there is no technical reason to exclude any of the data sets, as they are truly independent in nature (different technique, different operators and different laboratory). Therefore, all four data sets were retained for the calculation of an overall mean value.

### Estimation of the uncertainty of the mean value for PET in the salt carrier RM

The mean value of the four data sets with its expanded uncertainty was 0.306 ± 0.058 mg of PET in the salt carrier as shown in Fig. 1. The additional measurements reported in this work resulted in a narrower band of confidence, which falls well within the previously established indicative range of 0.293 ± 0.081 mg PET [9]. The solid red line shows the mean value whereas the dashed red lines show the expanded uncertainty. By applying the approaches laid down in ISO Guide 35:2017 [13], the expanded uncertainty of the mean value was calculated applying Eq. 2 [13]. The specific contribution for homogeneity (HOM) of 7.9% was calculated from ten measurements using ^1^H-NMR at JRC-Geel. For the homogeneity assessment, an approach described by Linsinger et al. was applied. No classical setup based on within- and between-bottle heterogeneity calculated by ANOVA could be used as subsampling is precluded [20]. For that reason, the method repeatability of ^1^H-NMR was subtracted from the observed standard deviation obtained for ten units measured under repeatability conditions. For characterisation (CHAR), the standard error of the unweighted mean based on the four data sets resulted in an uncertainty contribution of 4.3%. For the long-term stability (LTS), an uncertainty contribution for 1 year was calculated. Repeated weighings over a period of 31 months were used to calculate a specific uncertainty contribution of 2.9% for 12 months of storage. In practice, three time-points for the mass of PET were available at 0, 10 and 31 months. Direct weighings of PET from the salt carrier were performed. No statistically significant trend could be observed, but the spread of the data gave a relative uncertainty of 2.9% projected over 1 year of storage using the approaches described in ISO Guide 35 [13].

For the homogeneity assessment using ^1^H-NMR, it was unfortunately not possible to select sample units covering the whole filling sequence, as units with lower fill-order numbers were exhausted. Instead, information from the homogeneity estimate described in previous work and new weighing data suggested that the obtained homogeneity of 7.9% is realistic [9].

2 $${u}_{\mathrm{CRM}}=\sqrt{{u}_{\mathrm{char}}^{2}+{u}_{\mathrm{hom}}^{2}+{u}_{\mathrm{lts}}^{2}}$$

**Fig. 3:**
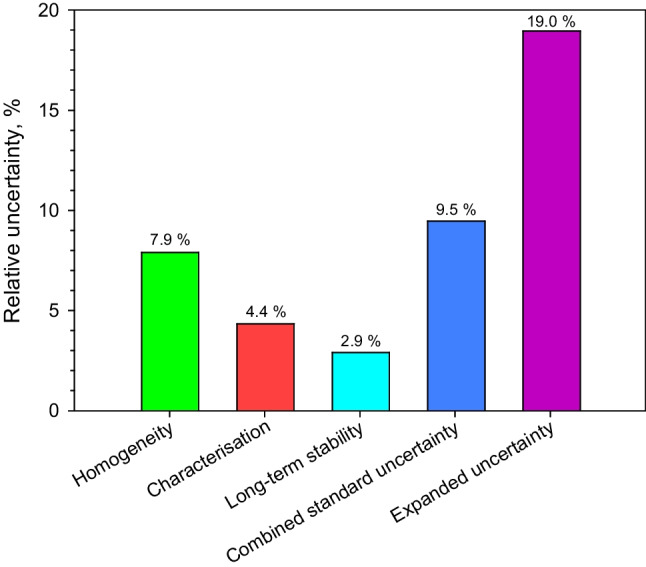
Homogeneity, characterisation and long-term stability uncertainties contribute to the standard uncertainty of the mean value that was calculated using Eq. 2. The expanded uncertainty of 19% was obtained by multiplying the relative standard uncertainty of 9.5% with a *k*-factor of 2 providing a confidence level of about 95%

All contributions to the standard uncertainty calculated using Eq. 2 are shown graphically in Fig. 3. An expanded uncertainty of 19% was obtained by multiplying the standard uncertainty of 9.5% with a coverage factor (*k* = 2) to provide a confidence level of about 95%. An expanded relative uncertainty around 20% is relatively large but not excessive in comparison with specific parameters in finalised CRMs with relatively high expanded uncertainties like ERM-CZ110 (ions in PM2.5-like atmospheric dust) and ERM-BC706 (pesticides in wheat flour) [21–23]. The expanded uncertainty obtained shows the potential of the approaches described here and in previous work for future productions of CRMs for microplastics [9]. All the necessary elements to determine an accurate mean value and its uncertainty for microplastics in the salt carrier were thus assessed and accounted for.

Both measurement techniques provide measurement results traceable to the kg whereby a statement about metrological traceability of the measurement results can be made which is also prerequisite necessary for future production of MP CRMs [8]. All measurement results can be found in Table S3.

## Conclusions

This report describes a first demonstration of good comparability between two independent data sets of ^1^H-NMR results and two independent data sets based on weighing using ultra-microbalances for a specific amount of PET MP in an RM. These measurement techniques will be applied to future production of MP CRMs. The methodology of preparing microplastic reference materials in a salt carrier that are further characterised in this way provides a good basis for production of calibrants or standards of MP that can subsequently be spiked to different matrices for trueness checks during method validation [9]. This possibility has also been shown here by adding the salt carrier RM containing PET to water, which simulates MP in a drinking water sample. Spiking offers the possibility of preparing RMs for MP in biological matrices or sediment as well. Such spiked matrices are more complex and can be used for further development of quantitative sample preparation techniques.

## Supplementary Information

Below is the link to the electronic supplementary material.

Supplementary file1 (DOCX 3049 KB)

## References

[1] Ritchie H, Roser M. Plastic pollution. https://ourworldindata.org/plastic-pollution. Accessed 11 Oct 2022.

[2] Frias JPGL, Nash R (2018). Microplastics: finding a consensus on the definition. Mar Pollut Bull.

[3] Leslie HA, van Velzen MJM, Brandsma SH, Vethaak AD, Garcia-Vallejo JJ, Lamoree MH. Discovery and quantification of plastic particle pollution in human blood. Environ Int. 2022;163:107199. 10.1016/j.envint.2022.107199. 10.1016/j.envint.2022.107199 35367073

[4] Liu S, Guo J, Liu X, Yang R, Wang H, Sun Y, Chen B, Dong R. Detection of various microplastics in placentas, meconium, infant faeces, breastmilk and infant formula: a pilot prospective study. Sci Total Environ. 2023;854:158699. 10.1016/j.scitotenv.2022.158699. 10.1016/j.scitotenv.2022.158699 36108868

[5] Jenner LC, Rotchell JM, Bennett RT, Cowen M, Tentzeris V, Sadofsky LR. Detection of microplastics in human lung tissue using μFTIR spectroscopy. Sci Total Environ. 2022;831:154907. 10.1016/j.scitotenv.2022.154907. 10.1016/j.scitotenv.2022.154907 35364151

[6] International Organization for Standardization. ISO/IEC 17025:2017 General requirements for the competence of testing and calibration laboratories. 2017. https://www.iso.org/publication/PUB100424.html

[7] Magnusson B Örnemark U (eds.). Eurachem guide: the fitness for purpose of analytical methods – a laboratory guide to method validation and related topics. (2nd ed. 2014). ISBN 978-91-87461-59-0. Available from https://www.eurachem.org/index.php/publications/guides/mv#download

[8] ISO Guide 30: Reference materials — selected terms and definitions. https://www.iso.org/standard/46209.html. Accessed 11 Oct 2022.

[9] Seghers J, Stefaniak EA, La Spina R, Cella C, Mehn D, Gilliland D, Held A, Jacobsson U, Emteborg H (2022). Preparation of a reference material for microplastics in water—evaluation of homogeneity. Anal Bioanal Chem.

[10] European Commission, Joint Research Centre, Belz S, Bianchi I, Cella C, Emteborg H, Fumagalli F, Geiss O, Gilliland D, Held A, Jakobsson U, La Spina R, Mehn D, Ramaye Y, Robouch P, Seghers J, Sokull-Kluettgen B, Stefaniak E, Stroka J. Current status of the quantification of microplastics in water: results of a JRC/BAM interlaboratory comparison study on PET in water. Publications Office, 2021, EUR 30799 EN, Publications Office of the European Union, Luxembourg; 2021. https://data.europa.eu/doi/10.2760/27641.

[11] International Organization for Standardization. ISO 17043: conformity assessment — general requirements for proficiency testing. 2010. https://www.iso.org/standard/29366.html. Accessed 12 Nov 2020.

[12] International Organization for Standardization. ISO 17034: general requirements for the competence of reference material producers. 2016. https://www.iso.org/standard/29357.html. Accessed 11 Oct 2022.

[13] International Organization for Standardization. ISO Guide 35: reference materials — guidance for characterization and assessment of homogeneity and stability. 2017. https://www.iso.org/standard/60281.html. Accessed 11 Oct 2022.

[14] Peez N, Becker J, Sonja M, Ehlers SM, Fritz M, Fischer CB, Koop JHE, Winkelmann C, Imhof W (2019). Quantitative analysis of PET microplastics in environmental model samples using quantitative ^1^H-NMR spectroscopy: validation of an optimized and consistent sample clean-up method. Anal Bioanal Chem.

[15] Peez N, Janiska MC, Imhof W (2019). The first application of quantitative ^1^H-NMR spectroscopy as a simple and fast method of identification and quantification of microplastic particles (PE, PET, and PS). Anal Bioanal Chem.

[16] Peez N, Imhof W (2020). Quantitative 1H-NMR spectroscopy as an efficient method for identification and quantification of PVC ABS and PA microparticles. Analyst.

[17] MestReNova (14.1.1 – 24571) Mestrelab Research S.L.. Santiago de Compostela, Spain. 2019. https://mestrelab.com/download/mnova/. Accessed 23/10/2022.

[18] ASTM D1193–06. Standard specification for reagent water. 2018. https://www.astm.org/Standards/D1193.htm. Accessed 17/01/2023.

[19] https://en.wikipedia.org/wiki/Tukey%27s_range_test. Accessed 24/11/2022.

[20] van der Veen A, Linsinger T, Pauwels J. Uncertainty calculations in the certification of reference materials. 2. Homogeneity study. Accred Qual Assur. 2001;6:26–30. 10.1007/s007690000238.

[21] CERTIFICATION REPORT. The certification of water-soluble ions in a fine dust (PM2,5-like) material: ERM-CZ110, EUR 30359 EN, 2020. https://crm.jrc.ec.europa.eu/p/q/cz110/ERM-CZ110-FINE-DUST-PM2-5-like-extractable-ions/ERM-CZ110. Accessed 19/10/2022.

[22] Charoud-Got J, Emma G, Seghers J, Tumba-Tshilumba MF, Santoro A, Held A, Snell J, Emteborg H. Preparation of a PM2.5-like reference material in sufficient quantities for accurate monitoring of anions and cations in fine atmospheric dust. Anal Bioanal Chem. 2017;409:7121–7131. 10.1007/s00216-017-0670-6. 10.1007/s00216-017-0670-6 PMC5717123 28971237

[23] CERTIFICATION REPORT. The certification of the mass fraction of pesticides in wheat flour: ERM-BC706, EUR 30972 EN, 2022. https://crm.jrc.ec.europa.eu/p/q/erm-bc706+/ERM-BC706-WHEAT-FLOUR-pesticides/ERM-BC706. Accessed 19/10/2022.

