# Supplementary material for: Feasibility of using quantitative 1H-NMR spectroscopy and ultra-microbalances for investigation of a PET microplastic reference material
Source: Anal Bioanal Chem. 2023 Feb 8;415(15):3033–40. doi: 10.1007/s00216-023-04567-0 (PMC10284926; doi:10.1007/s00216-023-04567-0)

**Table S1:** Figures of merit for ^1^H-NMR data from the University of Koblenz. Coefficient of correlation and limits of detection and quantification using external calibration of the aliphatic PET signal as well as the aromatic PET signal. Further below, masses and Relative Standard Deviation, % RSD (n=3) for five PET RM samples determined by ^1^H-NMR and the external calibration approach.

|  |  | **Calibration** | | |  | **RM Samples** | |
| --- | --- | --- | --- | --- | --- | --- | --- |
|  |  | **R²** | **LOQ [µg/mL]** | **LOD [µg/mL]** |  | **m [µg]** | **RSD, %** |
| PET_aliphat._ |  | 0.99989 | 6.09 | 1.83 |  | 298.74 | 7.26 |
| PET_arom._ |  | 0.99991 | 3.31 | 0.99 |  | 301.76 | 6.94 |

| **Sample Nr.** | | **411** | **412** | **413** | **419** | **422** |
| --- | --- | --- | --- | --- | --- | --- |
| PET_aliphat._ | m[µg] | 272.85 | 332.13 | 293.52 | 303.42 | 291.77 |
|  | RSD_individual_ | 0.64 % | 3.50 % | 0.58 % | 0.77 % | 0.64 % |
| PET_arom._ | m[µg] | 275.88 | 332.26 | 304.32 | 306.01 | 290.31 |
|  | RSD_individual_ | 1.49 % | 1.13 % | 0.23 % | 2.67 % | 1.32 % |

**Table S2:** Figures of merit for ^1^H-NMR data for MP PET in a NaCl-matrix (JRC-Geel). Quantitative data obtained by internal calibration using DMSO_2_ and the aliphatic PET signal. Repeatability and intermediate precision %-RSDs was obtained by measuring four PET samples in a NaCl- matrix by ^1^H-NMR on two different days (n = 8) and then calculated by Analysis of Variance, ANOVA.

| **Parameter** | **Result** |
| --- | --- |
| Repeatability precision | 4.9 % |
| Intermediate precision | 10.2 % |
| Over all recovery of PET from NaCl-matrix (n = 8) | 77 ± 5 % |
| Relative expanded measurement uncertainty, k = 2 | 23 % |

**Table S3.** All measurement results obtained on the PET reference material using ^1^H-NMR and two different ultra-microbalances.

| Vial number (fill sequence) | Mass of PET, mg  ultra-micro balances, JRC-Geel | Mass of PET, mg  ^1^H-NMR, JRC-Geel^b^ | Mass of PET, mg ^1^H-NMR,  Uni-Koblenz^c^ |
| --- | --- | --- | --- |
| 6 | 0.297 |  |  |
| 45 | 0.350 |  |  |
| 92 | 0.242 |  |  |
| 132 | 0.320 |  |  |
| 177 | 0.342 |  |  |
| 222 | 0.261 |  |  |
| 258 | 0.323 |  |  |
| 288 | 0.341 |  |  |
| 359 | 0.257 |  |  |
| 389 | 0.237 |  |  |
| 411 |  |  | 0.273 |
| 412 |  |  | 0.332 |
| 413 |  |  | 0.294 |
| 418 | 0.277 |  |  |
| 419 |  |  | 0.303 |
| 422 |  |  | 0.292 |
| 432 |  | 0.369 |  |
| 434 | 0.315 |  |  |
| 441 | 0.304 |  |  |
| 446 |  | 0.353 |  |
| 458 |  | 0.340 |  |
| 459 |  | 0.405 |  |
| 466 | 0.239 |  |  |
| 474 |  | 0.311 |  |
| 476 |  | 0.323 |  |
| 478 |  | 0.393 |  |
| 479 |  | 0.300 |  |
| 481 | 0.251^a^ |  |  |
| 486 |  | 0.311 |  |
| 487 |  | 0.355 |  |
| 488 | 0.313^a^ |  |  |
| 503 |  | 0.368 |  |
| 504 | 0.293^a^ |  |  |
| 512 |  | 0.385 |  |
| 515 | 0.286^a^ |  |  |
| 517 |  | 0.363 |  |
| 518 |  | 0.246 |  |

a = Results obtained using slightly different filtration set-up, metal funnel and the UMT2 balance. b = Each measurement result has an relative expanded measurement uncertainty of 23 %, U, which was obtained in a separate method validation study (see Table S2), c = Only the aliphatic protons signals in PET were used to obtain these results.

**Figure S1** From the left; an RM vial with salt carrier containing PET, filter holder with glass frit, rubber ring, 1 x 1 cm silicon membrane filter and the custom-made metal funnels and 20 ml glass recipient used for filtration of the RM samples.


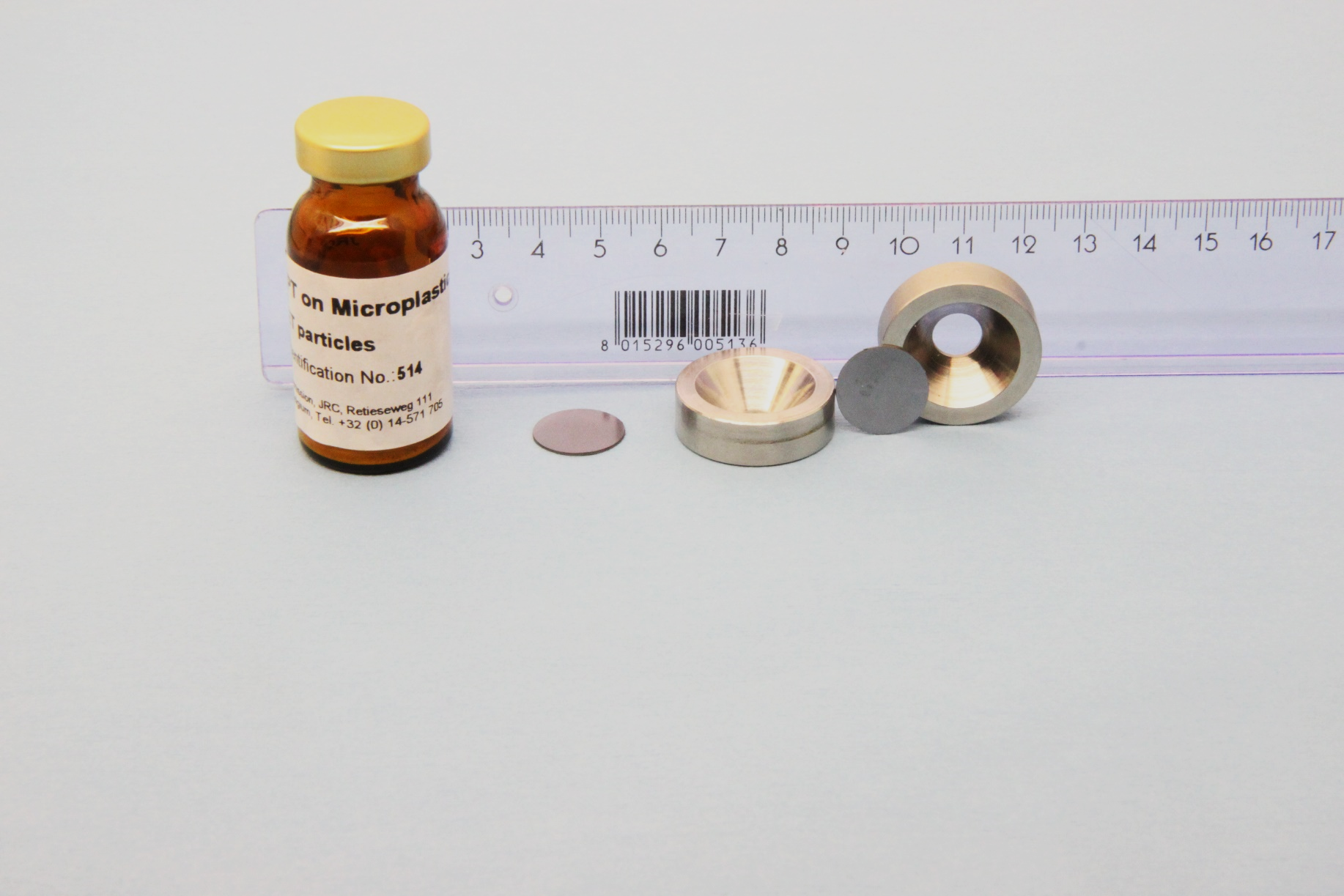

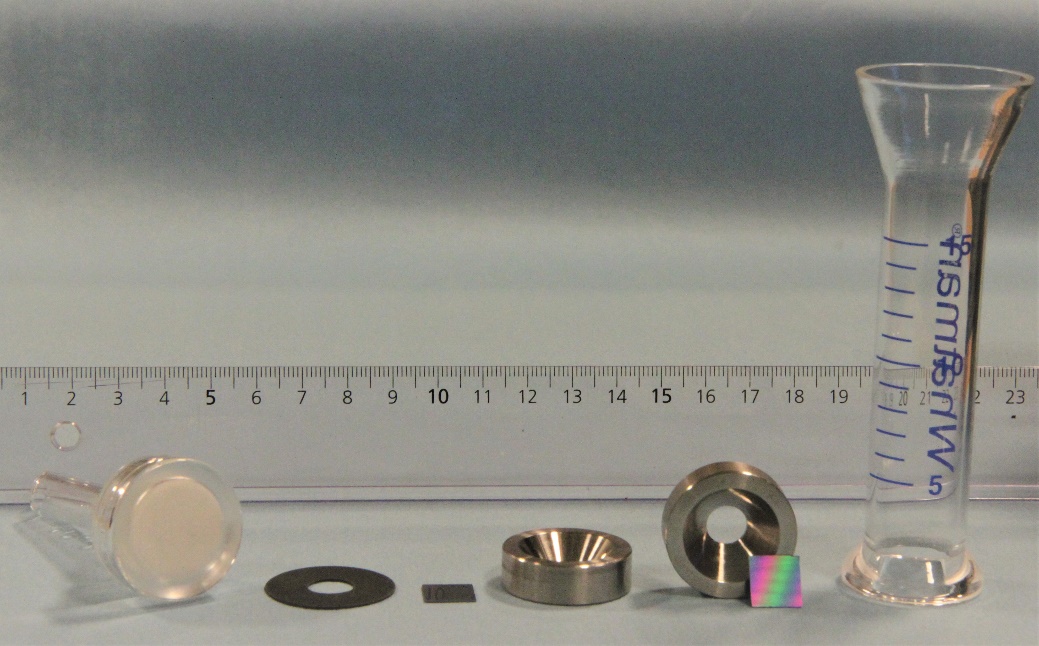


**Figure S2** ^1^H-NMR spectra (University of Koblenz). Comparison of PET RM sample containing DMSO and HMDSO as internal standards (top). PET RM sample without any internal standard (middle) and PET reference spectra containing HMDSO as INST (bottom),
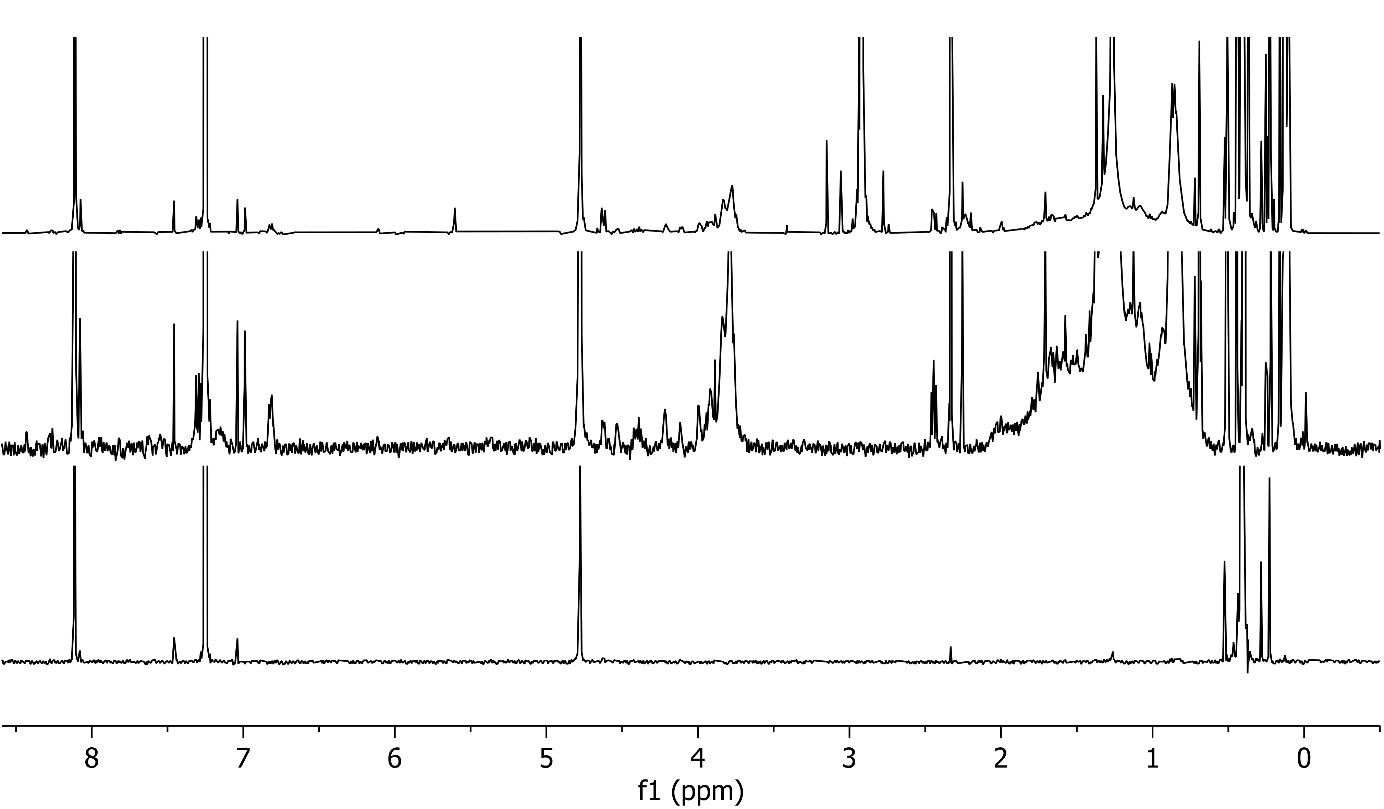

Supplement: Supplementary file 1 — Supplementary file1 (DOCX 3049 KB) [file 216_2023_4567_MOESM1_ESM.docx]
